# Interprofessional Curriculum Design: From Conception to Delivery

**DOI:** 10.1093/geroni/igaf122.3482

**Published:** 2025-12-31

**Authors:** Lisa Bodenheimer, Anne Rosenfeld, Stephanie Berdugo-Hernandez

**Affiliations:** Rowan University, Glassboro, New Jersey, United States; Rowan University, Glassboro, New Jersey, United States; Rowan Virtua School of Osteopathic Medicine, Stratford, New Jersey, United States

## Abstract

With funding awarded through the DHHS-HRSA Geriatric Workforce Enhancement Program Grant, the Rowan-Virtua School of Osteopathic Medicine (RV-SOM) has partnered with the Rowan University- Masters in Social Work Program (RMSW) to develop an interprofessionally designed and delivered post-graduate certificate in Geriatrics and Gerontology. RMSW is a new graduate program launched in 2023 and currently has no elective courses in aging. In 2024, initial pre-planning meetings were convened between both departments to conceptualize the components necessary to train the future workforce in geriatric and gerontology best practices with a specific focus on Alzheimer’s Disease and Related Dementia. A (5) course elective program was conceived to be offered to master level students university-wide, not just RMSW matriculated students, aiming to recruit a diverse target audience including students in non-clinical programs. Once complete, the (5) course program will be offered as a post-graduate certificate. During AY 24/25, a work group began syllabus development for the first elective course, “Introduction to Geriatrics and Gerontology”. The work group has a tripart mission; (1) to obtain stakeholder buy-in from multiple disciplines to co-develop modules from their scope of practice, (2) obtain buy-in from those practitioners to teach in the graduate program, allowing students the opportunity to learn directly from multiple disciplines and (3) create a syllabus which merges theory with practical approaches and interventions. To date, we have successfully engaged high-level faculty in geriatrics, neuropsychology, social work, and occupational therapy. All disciples will be engaged in didactics, small group activities, reading assignments and evaluation.

